# Auto-generated database of semiconductor band gaps using ChemDataExtractor

**DOI:** 10.1038/s41597-022-01294-6

**Published:** 2022-05-03

**Authors:** Qingyang Dong, Jacqueline M. Cole

**Affiliations:** 1 Cavendish Laboratory, University of Cambridge, J. J. Thomson Avenue, Cambridge, CB3 0HE UK; 2 ISIS Neutron and Muon Source, Rutherford Appleton Laboratory, Harwell Science and Innovation Campus, Didcot, Oxfordshire OX11 0QX UK; 3 Department of Chemical Engineering and Biotechnology, University of Cambridge, West Cambridge Site, Philippa Fawcett Drive, Cambridge, CB3 0AS UK

**Keywords:** Electronic and spintronic devices, Semiconductors, Electronic devices, Semiconductors

## Abstract

Large-scale databases of band gap information about semiconductors that are curated from the scientific literature have significant usefulness for computational databases and general semiconductor materials research. This work presents an auto-generated database of 100,236 semiconductor band gap records, extracted from 128,776 journal articles with their associated temperature information. The database was produced using ChemDataExtractor version 2.0, a ‘chemistry-aware’ software toolkit that uses Natural Language Processing (NLP) and machine-learning methods to extract chemical data from scientific documents. The modified Snowball algorithm of ChemDataExtractor has been extended to incorporate nested models, optimized by hyperparameter analysis, and used together with the default NLP parsers to achieve optimal quality of the database. Evaluation of the database shows a weighted precision of 84% and a weighted recall of 65%. To the best of our knowledge, this is the largest open-source non-computational band gap database to date. Database records are available in CSV, JSON, and MongoDB formats, which are machine readable and can assist data mining and semiconductor materials discovery.

## Abstract (summary)

**Table:** 

Measurement(s)	semiconductor band gaps
Technology Type(s)	natural language processing

## Background & Summary

Semiconductors have been at the heart of materials science and the electronics industry. There are increasing demands for semiconductors to be used in solar panels, transistors, light-emitting diodes, and so forth, given their function depends upon the energy difference between the conduction band minimum and valence band maximum of a material, i.e., the band gap. Data on these band gaps have therefore gained significant importance and interest. If assembled in structured and organized forms, these data not only allow researchers to perform background research with little effort, saving valuable time in literature reading and avoiding unnecessary experiments; they also enable the possibility of data visualization and mining applications to predict unknown materials and their properties, hence accelerating materials discovery^1,2^.

Not unlike other fields in physics, such as high-energy physics and astronomy^3,4^, there are several large-scale big-data projects in the domain of materials physics. For example, the Materials Genome Initiative^5^, the Materials Project^6^, and the Automatic FLOW for Materials Discovery (AFLOW)^7–9^ all aim to generate databases of materials properties using electronic-structure calculations and build a software framework for accelerated materials discovery. However, nearly all current large-scale semiconductor databases use high-throughput computational techniques to cover a wide grid of parameter space for semiconductors. The involved computational methods vary significantly in theories and software execution, making it impractical to compare numbers across different databases. Also, the calculated properties, although large in quantity (i.e. tens of thousands of data records), are rarely confirmed by experiments, which makes it difficult to examine their data quality. In contrast, databases of experimental band gaps are comparably smaller in size, typically only several thousand data records; see for example, the BandgapDB^10^.

These drawbacks of computationally-generated semiconductor databases have led to interest in the curation of experimental data from the scientific literature using Natural Language Processing (NLP) techniques^11^. In combination with computationally-generated databases, NLP-based databases provide several additional benefits. First is the quality control of databases, by comparing pairwise experimental and computational data of a given material; highly discrepant values would indicate questionable data quality in at least one of these databases, while high consistency likely suggests good data accuracy. Second is that a good match between experimental and computational data can verify the correctness of the approximations and wave functions used in the source ab-initio calculations, making the computational databases more reliable. Third is the possibility of exploring new materials and predict future research trends by applying machine-learning techniques to the databases^12,13^.

Unfortunately, generating materials databases using NLP techniques presents many challenges. The information about material properties is highly fragmented and unstructured owing to the diversity, variation, and ambiguity in natural language. Authors may present their findings in different sections of a document such as body texts, tables, and figures. The same information can also be written in different ways due to inconsistent terminologies that are used across various material categories, making it difficult for generic NLP toolkits to balance accuracy and generalizability. Moreover, the exponentially growing number of publications^14^ makes it impossible to extract data and annotate data manually. Therefore, it is crucial that the data-extraction process is fully automated and requires little human intervention. Several NLP toolkits that can parse and extract chemical information from scientific documents have been made openly available, such as ChemicalTagger^15^, Chem Spot^16,17^, LeadMine^18^, and OSCAR4^19^; although, many of them have been designed to handle only specific sections of a scientific paper.

ChemDataExtractor^20,21^ is a Python-based software toolkit that uses NLP and machine-learning methods to extract chemical information from all sections of a scientific document. This work presents an automatically generated database of 100,236 semiconductor band gap records using ChemDataExtractor version 2.0. Temperature information, nested to corresponding band gap records, were also extracted and included in the database. All data records were extracted from a corpus of 128,776 journal articles using both the NLP pipeline within ChemDataExtractor, and the modified Snowball algorithm^22^, which is a semi-supervised machine-learning method, incorporated into ChemDataExtractor version 2.0. This modified Snowball algorithm has been extended in this study to incorporate nested property models, where the parent property is dependent on the nested properties. Its hyperparameters have also been optimized so that it complies with the NLP pipeline to afford data extraction with optimal performance.

## Methods

### Text acquisition

The first stage in the automatic curation of data from literature is to acquire a corpus of relevant articles that contain semiconductor band gap information. For this database, the journal articles were sourced from three publishers, namely Elsevier, the Royal Society of Chemistry (RSC), and Springer. These journal papers are best suited for an NLP workflow for several reasons. They could be downloaded and stored locally; they are digital rather than scanned, at least while they are sufficiently recent, so they can be processed without introducing errors by having to convert images into characters; and they are traceable by DOIs so that data extracted from them can be referenced.

RSC papers were downloaded using the web-scraping tools built into ChemDataExtractor 2.0. These functions allow a query text “band gap” to be submitted to the search field of the RSC homepage in a repeatable way. The response in each page was recorded, and the article metadata, which contain information such as title, author names, URL link, and DOI, were extracted from the responses. The contents of the relevant papers were accessed by their DOIs or URLs, if provided, where they were written in hypertext markup language (HTML). A total of 63,358 papers were downloaded.

Elsevier papers were downloaded via the ScienceDirect Search API V2, which is the Application Programming Interface (API) provided by Elsevier for text and data mining purposes. Article metadata were first extracted from the response of the Elsevier server and contents were then accessed where written as extensible markup language (XML). A restriction on the publication year (2005–2020) was imposed when requesting metadata from Elsevier. This is because an increasing portion of earlier published papers were found to be scanned from images, and then converted into UTF-8 characters; thus, they contain a large number of errors on the text level, which lower the accuracy of the data significantly. A total of 24,057 papers were downloaded.

Similarly, Springer papers were downloaded via the Text and Data Mining API, which allows access to papers that are not openly available. Responses from the Springer server contain the full content of papers in Journal Archiving and Interchange Tag Set (JATS) format, and the contents were then retrieved in this format. A total of 41,361 papers were downloaded.

It is worth mentioning that during this text acquisition stage, no articles in portable document format (PDF) were downloaded, which is one of the most common formats in scientific publications. This is primarily because many relatively old PDFs may be preserved as images or contain sections that are scanned from printed papers, as seen in some of the early papers from Elsevier. Converting PDF to plain text is still an active area of research, and inevitably introduces an uncontrollable amount of error into the NLP pipeline.

### Document processing

The second stage is to standardize and normalize articles. This is to eliminate any inconsistencies amongst different file formats and simplify the parsing rules in the phrase parsing stage. First, the HTML/XML files were converted into plain text using the “reader” package in ChemDataExtractor. The JATS files from Springer were processed by first stripping them of their tag pairs which represent the hierarchical information and metadata of an article. The remaining contents were then split into individual paragraphs of plain text.

The plain text of all articles was then processed using the NLP pipeline of ChemDataExtractor 2.0, as described in^20^. ChemDataExtractor provides comprehensive NLP functions such as sentence splitting, tokenization, word clustering, part-of-speech (POS) tagging, and chemical named entity recognition (CNER). Previous studies based on ChemDataExtractor have successfully produced databases of magnetic properties^22^, battery materials^23^, and UV absorption spectra^24^. The output of a processed article file is a document object, which is an ensemble of structured sentence objects, each represented by a list of text tokens that are tagged by their syntactic functions to further facilitate phrase parsing.

### Phrase parsing and relationship extraction

The third stage, phrase parsing, is the most challenging problem in the NLP pipeline due to high level of ambiguity and implicit knowledge carried within natural language. One example NLP toolkit, ChemicalTagger^15^, uses a universal rule-based grammar for parsing certain sections of papers in the chemistry domain. The default parser of ChemDataExtractor 2.0, AutoSentenceParser, employs a similar approach, using multiple specialized grammar rules that have been designed to extract more specific types of chemical information. A grammar rule is composed of a series of nested rules, written as regular expressions, that translate a list of tagged text tokens into a tree model, which describes the syntactic structure of a sentence. A relationship can thus be extracted based on the tree structure of a sentence. This work uses AutoSentenceParser to parse and extract band gap information from articles with few modifications, except for defining a nested property model, which is partly inherited from pre-defined unit models in ChemDataExtractor.

However, when generalized from chemistry to the wider materials science, these grammar-based parsing rules used in AutoSentenceParser become less efficient. As more writing styles and complexity are introduced into the article corpus, the number of grammar rules grows rapidly in order to maintain the same level of accuracy, not to mention that a large amount of testing is required to generate successful and efficient rules. This is a major barrier that limits the performance of rule-based phrase parsing in terms of accuracy. Moreover, the difficulty of writing new grammar rules also increases when expanding their use cases. Data extraction on sentences that contain only slight deviations from the grammar rules will fail. Therefore, the grammar rules need to be both specific enough to maintain accuracy, and general enough not to miss too much information.

These limitations of completely deterministic phrase parsing motivated the use of a probabilistic method. The modified Snowball algorithm^22,25^, also built into ChemDataExtractor 2.0, is such a method that allows variations between text and parsing rules, and generates a confidence score based on the degree of variation or similarity. The extracted data record can then have a parameter, the minimum confidence threshold ${\tau }_{c}$, that measures the likelihood of it being correct. This is a feature that is not available in AutoSentenceParser or any other deterministic phrase parsing method.

### The modified Snowball algorithm

As with other semi-supervised machine learning algorithms, the first stage of the Snowball pipeline is to acquire a set of labeled data. These data are used to train a Snowball model by manually analyzing and curating a set of extraction patterns from training sentences. 850 training articles, which were randomly chosen from the entire article corpus, were split into sentences, and then tokenized into lists of word tokens. A sentence is considered to be a candidate if all entities of a complete relationship tuple, namely a chemical name, a property specifier, a property value, and a property unit, can be found in the token list. Each list is then divided into three elements: prefix, middle, and suffix, which are defined by their positions relative to the relationship entities. These three elements, together with relationship entities, form the basis of a phrase object; they are then vectorized and each are assigned a normalized weight. Further details about the objects of the Snowball model with examples are given in Table 1.

**Table 1:** Descriptions and examples of Snowball objects.

Object name	Description *Example*
Sentence	Sentence split from body text. *“The bulk TiO2 has a direct band gap of 3.2 eV at tau point.”*
Relationship tuple	An entity list made of chemical name, property specifier, property value, and property unit. *[TiO2, band gap, 3.2, eV]*
Prefix	Word tokens before the first entity. *[“The”, “bulk”]*
Middle	Word tokens between each relationship entity. *[“has”, “a”, “direct”, “of”]*
Suffix	Word tokens after the last entity. *[“at”, “tau”, “point”]*
Weight	Normalized importance factor for prefix, middles, and suffix. *(0.1, 0.8, 0.1)*
Phrase object	A normalized vector of entities. *[“The bulk”, chemical name, “has”, “a”, “direct”, specifier, “of”, value, unit, “at tau point”]*

To improve model efficiency, all phrase objects found in training sentences were clustered according to a two-level hierarchy. The first level separates phrases based on the number and ordering of relationship entities, where phrases with the same number and sequence of relationship entities are assigned to the same main cluster. The second level is based on the similarity between two phrases, defined by

1 $$sim\left(p,q\right)=\mathop{\sum }\limits_{i=1}^{m+1}{v}_{i}\left({p}_{i}\cdot {q}_{i}\right),$$

where *p* and *q* are two phrase objects, $\left({p}_{i}\cdot {q}_{i}\right)$ is a dot product of two vector elements, ${v}_{i}$ is a normalized importance weight vector, and *m* is the number of relationship entities. The similarity value ranges from 0% to 100%, with 100% representing identical phrases. Within a main cluster of phrase objects, several sub-clusters are created. The first phrase is assigned to its own sub-cluster; a proceeding phrase is also assigned to this sub-cluster if its similarity to a combination of the common elements of the existing sub-cluster (its centroid extraction pattern) is equal to or above that of a predefined minimum similarity threshold, ${\tau }_{sim}$; if not, the phrase is assigned to a new sub-cluster. During training, ${\tau }_{sim}$ was set to the relatively high value of 90%. The motivation behind this setting is not to achieve best performance of the Snowball model, but is to generate as many sub-clusters as possible, which is the primary factor that affects data extraction.

Other than phrases, two more attributes are used to characterize a sub-cluster: the centroid extraction pattern, and a confidence value *C(P)*, which describes the likelihood that the centroid pattern *P* produces correct relationships. By comparing the centroid extraction pattern with all training sentences, the confidence value is the fraction of correct relationship tuples amongst all extracted tuples:

2 $$C\left(P\right)=\frac{{\rm{Number}}\;{\rm{of}}\;{\rm{positive}}\;{\rm{matches}}}{{\rm{Total}}\;{\rm{number}}\;{\rm{of}}\;{\rm{matches}}}.$$

In this work, about 500 band gap relationship tuples were manually extracted from 850 randomly selected training articles, and 400 sub-clusters were created. Table 2 shows an example of a sub-cluster.

**Table 2:** An example of a sub-cluster containing four phrases, selected from a trained Snowball model.

Components	Description
Phrase 1	This insulating Al2O3 has a wide band gap Eg of 7–9 eV and acts purely as a mesoporous scaffold for the perovskite (CH3NH3PbI2Cl) to be deposited.
Phrase 2	In addition, ZnO has a wide band gap of 3.37 eV, which inevitably restricts its practical application in visible light or sunlight.
Phrase 3	However, TiO_2_ has a wide band gap of 3.2 eV which limits its application under visible light.
Phrase 4	Pure TiO_2_ has a band gap of 3.2 eV and on loading CoOx, the band gap shifted to the visible region, as shown in Table 1.
Centroid extraction pattern	(compound_names) has a wide (bandgap_specifier) of (bandgap_raw_value) <Blank> (bandgap_raw_units)
Confidence	1.0

Once a Snowball model has been trained sufficiently, it acts as a phrase parser that can extract band gap relationships from new sentences. Each candidate relationship, ${r}_{c}$, derived from a candidate phrase, ${p}_{c}$, is also assigned a confidence score to reflect its likelihood of correctness. The confidence score $C({r}_{c})$ is defined as

3 $$C\left({r}_{c}\right)=1-\mathop{\prod }\limits_{i=1}^{n}\left[1-C\left({P}_{i}\right)\cdot sim\left({p}_{c},{P}_{i}\right)\right],$$

where ${P}_{i}$ are centroid extraction patterns, and *i* sums over all sub-clusters within a main cluster of phrase objects. This scoring scheme ensures that a candidate phrase which matches to more extraction patterns with high similarity has higher confidence score than those with fewer matches. Finally, if the confidence score is equal to or above that of a minimum confidence threshold ${\tau }_{c}$, the candidate relationship is accepted, and added to the best matching sub-cluster. It is worth noting that a bootstrapping loop, or positive feedback loop, is in effect, such that, during data extraction, unseen phrases are added into sub-clusters, and this new information can be reflected in the updated centroid extraction pattern. Thus the Snowball model can be understood as an active learning model, since it learns from previously unseen sentences and it is more likely to accept frequently occurring phrases, and improve in performance over time.

In this work, the Snowball algorithm has been extended in order to handle nested models, where the parent property is dependent on the nested properties, but the nested properties are not necessarily intrinsic characteristics of a material. For the band gap of semiconductors, we chose temperature as the nested property for two reasons: temperature heavily affects interionic spacings and electron-phonon coupling constants, and can thus have measurable impact on band gap values; and temperature information is easily representable by a value and a unit. In version 2.0 of ChemDataExtractor, a nested model is defined as a pair of property models that share the same chemical compound. A complete relationship can thus be either quaternary: chemical name, band gap specifier, energy value, energy unit; or septenary, where it can contain three optional entities: a temperature specifier, a temperature value, and a temperature unit. The same clustering method, confidence scoring mechanism, and relationship extraction process of the default modified Snowball algorithm still hold for nested models.

**Fig. 1:**
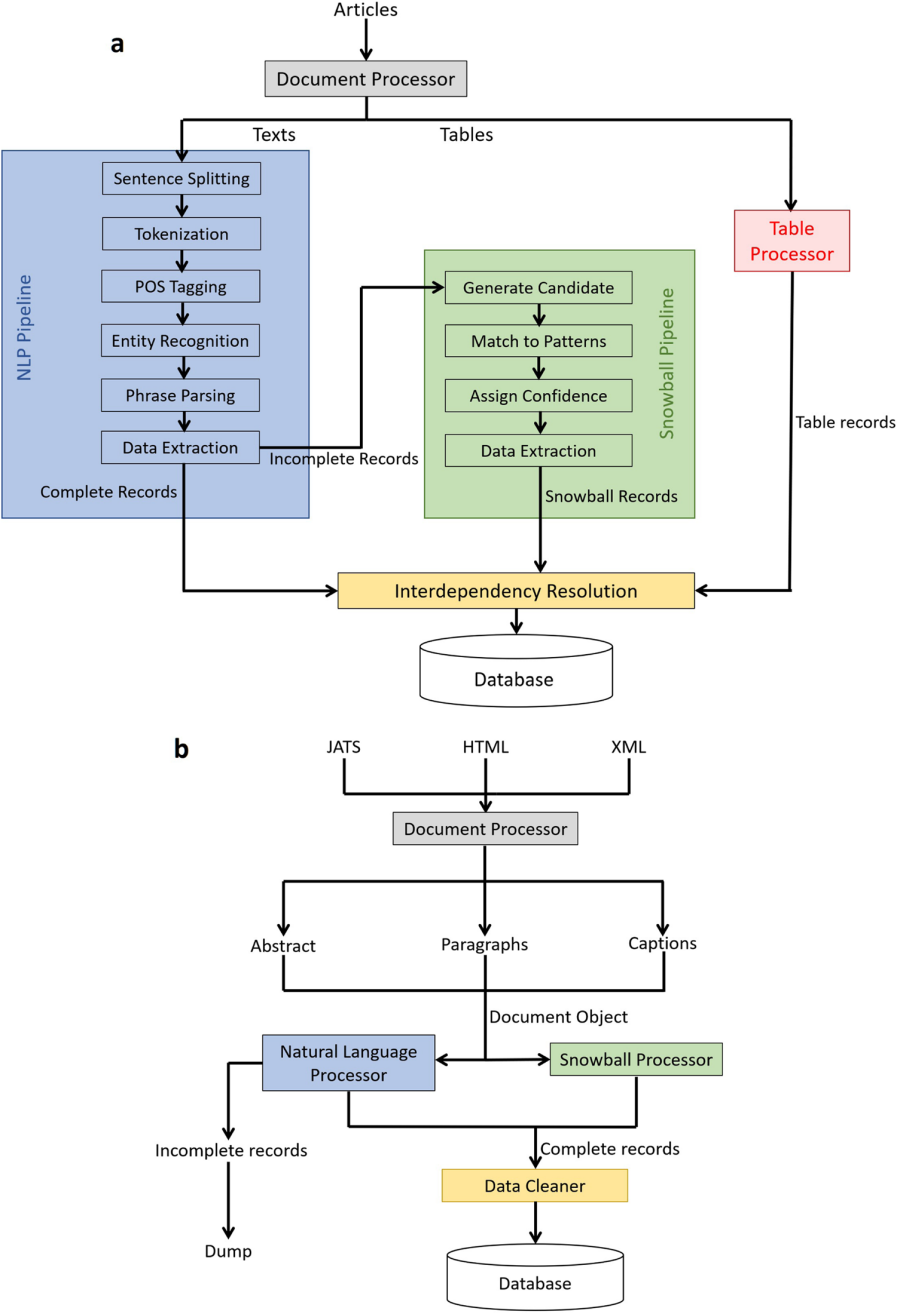
The general workflow of the original ChemDataExtractor version 2.0 (top), and the workflow adopted in this work (bottom).

Unlike the standard workflow of ChemDataExtractor version 2.0, where documents are processed by either AutoSentenceParser or the Snowball parser, sentences were fed into both parsers simultaneously in this work, as shown in Fig. 1. Identical records extracted by both parsers were combined during post processing, so that double counting could be avoided. We chose this approach because the relative importance of the Snowball parser amongst other parsers in ChemDataExtractor has changed due to the increase in performance realized through this work. By fine tuning the hyperparameters of the Snowball model, it shows much higher precision and similar recall compared with AutoSentenceParser, whereas it was initially introduced into ChemDataExtractor as a high-precision low-recall add-on to its NLP pipeline. More details are discussed in later sections of this paper.

### Post processing

The final stage of the data extraction process is to combine, normalize, and clean up raw data records in order to improve the quality and readability of the database. For completeness, all 7,656 records in BandgapDB^10^, a manually-curated experimental database, were also integrated into this database. The energy units of all data records were normalized to eV; temperature units in Celsius were converted to Kelvin. Entries that are exactly identical from AutoSentenceParser and Snowball were merged to avoid double counting. Each data record was then tagged with the sentence from which it was extracted, article DOI, and publisher from which the article was sourced. For more convenient querying, chemical names are also represented as compositions (elements and their corresponding numbers of atoms per molecule), by parsing the extracted chemical strings using MaterialParser^26^. A quick overview shows that 79% of all 21,053 unique chemical names can be parsed and represented by their compositions, which take up 87% of all data entries in the database. Chemical strings that cannot be parsed by MaterialParser are mostly classes of semiconductors or trade names, which do not have definitive chemical formulae. The correctness of these data records is unrelated to the missing composition space, and is evaluated in later sections.

For data cleaning, several additional rules were implemented to remove invalid records from the database. These rules were developed by manually examining the database and identifying common errors. For example, records with a temperature unit in Fahrenheit were removed since they incorrectly refer to specific capacitance in all examined cases; energy units expressed in Joule were removed for the same reason; energy units of keV and MeV were also removed since these values are several orders of magnitudes larger than the band gap of a typical inorganic compound and are most likely wrong; band gaps smaller than 0 eV or larger than 20 eV were removed since they are either invalid or out of the range of interest; certain chemical names or acronyms (i.e. “VB”, “VBM”, “VOC”, “oxygen”, “nitrogen”, etc.), chemicals that are purely elemental and are not amongst the seven elemental semiconductors (Ge, Sn, Si, Se, Te, S, and C), and those ending with “+” or “-” were removed as they are most likely dopants rather than semiconductors; and band gap values preceded by keyword “by” were removed since nearly all of these values refer to energy differences rather than the absolute band gap values. Evaluation on 200 of the raw data records shows that these data cleaning rules can improve the accuracy of the database by up to 10% for Snowball records and nearly 20% for AutoSentenceParser records.

To further improve the accuracy of the database, a custom Snowball model was trained using error-prone sentences that were identified when training the general Snowball model. These sentences typically contain phrases that yield invalid relationships which would be otherwise accepted by the general Snowball model. Relationships extracted by the special Snowball model were compared with all records in the database, and identical entries were deleted. A very high ${\tau }_{sim}$ of 95% was assigned to the custom Snowball to ensure a high level of accuracy. Otherwise, correct records in the database could be incorrectly deleted. Admittedly, setting ${\tau }_{sim}=100 \%$ would guarantee 100% accuracy, but the custom Snowball model will only accept pretrained phrases, making it equivalent to manual data cleaning, so a setting to 100% would defy the purpose of the custom Snowball model.

## Data Records

The database can be downloaded from *Figshare*^27^, and it is available in CSV, JSON and MongoDB formats. Table 3 provides an overview of the data records. *Name* is the string corresponding to the chemical compound in the original text. A single compound can sometimes have multiple identifiers within one sentence (i.e. *Titanium dioxide (*$Ti{O}_{2}$*) has a band gap of 3.2 eV*.), and each identifier is assigned to a separate data record. *Composition* is the composition space of the chemical formula given in pairs of elements and numbers. Chemical strings that cannot be parsed by MaterialParser have this field set to null. *Raw_value* and *Raw_unit* are text strings that have been extracted from the source document that indicate band gap values and units. These energy values and units are normalized into eV during post processing and given as *Value* and *Unit*. In many instances, one chemical compound can have two band gap values that indicate a range. Therefore, the *Value* field can be a list of one floating point number or a list of two floating point numbers. The same applies for *Temperature_raw_value*, *Temperature_raw_unit*, *Temperature_value*, and *Temperature_unit*, if a complete temperature record is found in a sentence and nested to a band gap record. If temperature information is not found, these four fields are set to null.

**Table 3:** Description of the band gap records and their attributes.

Key	Description	Data type
Name	Chemical compound names	String
Composition	Elements and their numbers of atoms per molecule	Dictionary
Value	Normalized band gap value	List of floats
Unit	Normalized band gap unit	String
Raw_value	Text string of band gap value	String
Raw_unit	Text string of band gap unit	String
Temperature_value	Normalized temperature value	List of floats
Temperature_unit	Normalized temperature unit	String
Temperature_raw_value	Text string of temperature value	String
Temperature_raw_unit	Text string of temperature unit	String
AutoSentenceParser	Source of the data record	Boolean
Snowball	Source of the data record	Boolean
BandgapDB	Source of the data record	Boolean
Confidence	Confidence score from Snowball model	Float
Text	Sentence from which data is extracted	String
Publisher	Name of the publisher of the paper	String
DOI	DOI of the paper	String
Notes	Additional reference for the data record	String

*AutoSentenceParser*, *Snowball*, and *BandgapDB* are Boolean tags that mark the source from which the data record is extracted. As the Snowball algorithm, AutoSentenceParser, and the BandgapDB have different level of precision and recall, these tags can provide a general estimate of correctness for data records. A record extracted by ChemDataExtractor can have both *AutoSentenceParser* and *Snowball* set to True, as identical records from both pipelines were combined during post processing. Similarly, a data record having *BandgapDB* set to True must have *AutoSentenceParser* and *Snowball* set to False. Additionally, a data record extracted by the Snowball pipeline has a *Confidence*, which is the confidence score assigned to this record using Eq. 3; otherwise, the *Confidence* field is set to null. All Snowball records were compiled into the database regardless of their confidence scores since the minimum confidence threshold is set to 0%, as described in previous sections. Information on the source articles are given in *Publisher*, *DOI*, and *Notes* field.

## Technical Validation

### Performance evaluation

The performance of the parsers was evaluated on the basis of precision, recall and F-score. Precision is the fraction of correct (relevant) records among all extracted records; recall is the fraction of successfully extracted records among all correct (relevant) records in the articles; and F-score is the harmonic mean of precision and recall, defined as

4 $$\begin{array}{l}Precision=\frac{TP}{TP+FP}\\ Recall=\frac{TP}{TP+FN}\\ F \mbox{-} score=2\cdot \frac{Precision\cdot Recall}{Precision+Recall}\end{array}$$

where *TP* is the true positive count (the number of records that are correctly extracted), *FP* is the false positive count (the number of records that are incorrectly extracted), and *FN* is the false negative count (the number of correct records that are not extracted).

**Fig. 2:**
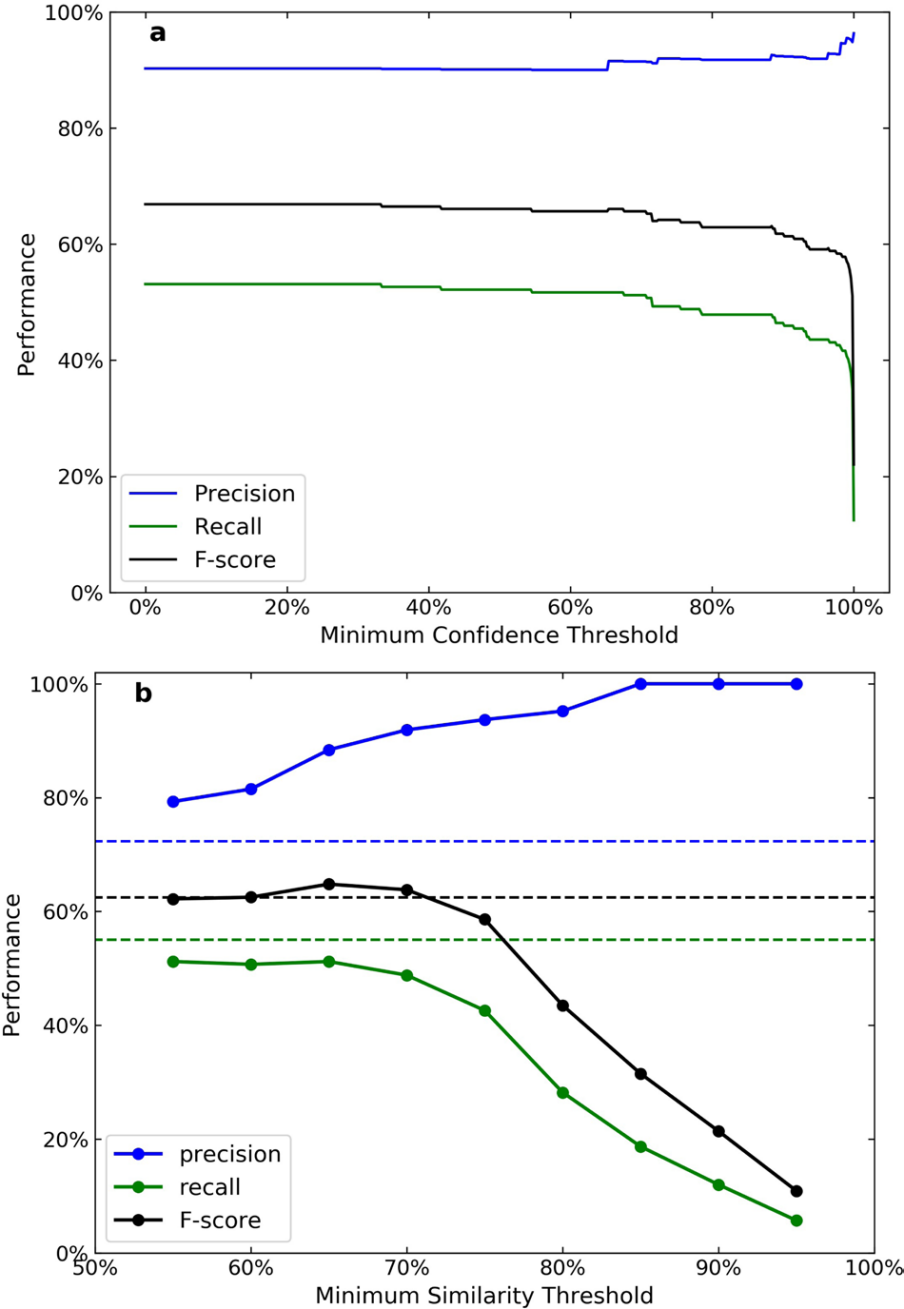
Performance of the Snowball parser and AutoSentenceParser against ${\tau }_{c}$ (**a**) and ${\tau }_{sim}$ (**b**), evaluated at dotted points. In (**a**), ${\tau }_{sim}$ is set to 65%; in (**b**), ${\tau }_{c}$ is set to 0%. For comparison, results of AutoSentenceParser are represented in dashed lines.

Annotating data records by TP, FP, and FN requires knowing all of the correct data records out of a set of articles. Therefore, 150 articles were randomly selected (50 from each publisher) to form the evaluation set; a total of 209 records were manually extracted from them. These same articles were then processed by AutoSentenceParser and the Snowball model separately, and the automatically extracted data records were compared with the manually annotated data. A record was counted as a true positive only if all entities in the relationship were present and correct. For example, a record with a correct chemical identification and a band gap property value and a unit but with a missing or incorrect temperature value and unit was considered to be a false positive. The evaluation results of AutoSentenceParser and the Snowball model are given in Table 4 and Fig. 2. Overall, AutoSentenceParser has a lower precision at 72%, which is below the typical 80% threshold error rate that is regarded for manual data extraction. This result is consistent with previous work with ChemDataExtractor, where the overall precision of the NLP pipeline was estimated to be between 66% and 73% for a magnetic materials database^22^, and around 80% for a battery materials database^23^ which were assisted by custom parsing rules.

**Table 4:** Evaluation results of AutoSentenceParser and the Snowball model.

Parser	${{\boldsymbol{\tau }}}_{{\boldsymbol{s}}{\boldsymbol{i}}{\boldsymbol{m}}}$	Total extracted	Removed	TP	Precision	Recall	F-score
AutoSentenceParser	N/A	212	53	115	72.3%	55.0%	62.5%
Snowball	95%	12	0	12	100.0%	5.7%	10.9%
Snowball	90%	25	0	25	100.0%	12.0%	21.4%
Snowball	85%	39	0	39	100.0%	18.7%	31.5%
Snowball	80%	67	5	59	95.2%	28.2%	43.5%
Snowball	75%	101	6	89	93.7%	42.6%	58.6%
Snowball	70%	123	12	102	91.9%	48.8%	63.8%
Snowball	65%	141	20	107	88.4%	51.2%	64.8%
Snowball	60%	157	27	106	81.5%	50.7%	62.5%
Snowball	55%	169	34	107	79.3%	51.2%	62.2%
Snowball	dynamic	147	21	113	89.7%	54.1%	67.5%

The fourth column “Removed” is the number of records that is deleted during post processing.

### Hyperparameter optimization

Before discussing the performance of the Snowball parser, it is worth highlighting one implicit parameter that has not been discussed by Court and Cole^22^. The learning rate, defined as the rate at which the Snowball model updates itself, controls the extent by which bootstrapping is employed in a Snowball model. A learning rate of 1 represents the normal workflow, whereby the phrases within a sub-cluster, the centroid extraction pattern, and its confidence value are all updated every time a new phrase is accepted. A learning rate of 0 fixes the confidence values of all centroid extraction patterns at a constant, whereas the centroid extraction patterns themselves are allowed to be updated. By default, the learning rate is set to 1. However, we found that by keeping the learning rate at 1 throughout training and data extraction yields poor recall. This is a reflection of the choice of hyperparameters and the clustering mechanism. During training, each phrase is assigned a confidence of 100%. And at a very high ${\tau }_{sim}=90 \%$, most of the phrases are assigned to their own sub-clusters, making the confidences of sub-cluster centroid extraction patterns very close to 100%. After training, more and more new phrases with lower confidence scores are added to the sub-clusters. Since most of the phrases are unique due to the complexity of natural language, a centroid extraction pattern starts to match to fewer phrases within a sub-cluster, making its confidence gradually decrease as the number of phrases increases, according to Eq. 2. Using Eq. 3, a lower centroid pattern confidence $C({P}_{i})$ means lower confidence scores for new candidate relationships. Hence, the Snowball model becomes increasingly unlikely to accept new phrases, even if the similarity between them and centroid patterns are high. This behaviour does not affect the precision of the Snowball model, but suppresses the recall significantly. Changing the learning rate to 0 after training completely solves this problem, which must be done manually. This ensures that the confidences of centroid extraction patterns remain at nearly 100% throughout data-extraction process, so that all new phrases with a good match can be equally likely to be accepted. Moreover, these new phrases are also added to the sub-clusters and the Snowball model can learn from them by updating only the centroid extraction patterns but not their confidences, so that the positive feedback loop is still in effect.

One advantage of the Snowball parser over AutoSentenceParser is the tuneable nature of a Snowball model. The two hyperparameters, ${\tau }_{sim}$ and ${\tau }_{c}$, can be optimized for either high precision or high recall. Intuitively, one would optimize ${\tau }_{c}$ for best performance, since the confidence score of a candidate relationship describes the likelihood of it being correct. Records with low confidence scores can be rejected even if they match to only few, if not none, of the centroid extraction patterns. However, we found that the F-score monotonically increases as ${\tau }_{c}$ is lowered from 100% to 0%, as shown in Fig. 2, indicating that setting ${\tau }_{c}$ to above 0% provides no meaningful performance improvement. Figure 2 also shows that while setting ${\tau }_{c}$ to a high value (>65%) will remove some of the incorrect records with low confidence scores, which will improve precision slightly, it will reduce recall drastically.

Another interesting observation is that when ${\tau }_{c}$ is lower than ${\tau }_{sim}$, which is optimized to 65% during evaluation, both precision and recall stay almost constant. Further inspection reveals that only 1% of all evaluation records have confidence scores below 65%, and ${\tau }_{c}$ fails to provide a meaningful performance difference in this region. This is, again, a result of the hyperparameter choices and the confidence scoring mechanism. During training, centroid extraction patterns have very high confidences at nearly 100%, and since the learning rate is set to 0 afterwards, the confidence values remain unchanged. If a candidate phrase matches to at least one of the centroid extraction patterns, most of the similarity values are close to 0% except for the matches. In the cases where there is only one match to a centroid extraction pattern *P*_*j*_, using approximation $C({P}_{i})\approx 1$, Eq. 3 reduces to

5 $$C\left({r}_{c}\right)\approx 1-\mathop{\prod }\limits_{i=1}^{n}\left[1-sim\left({p}_{c},{P}_{i}\right)\right]\approx 1-\left[1-sim\left({p}_{c},{P}_{j}\right)\right]=sim\left({p}_{c},{P}_{j}\right),$$

since $1-sim({p}_{c},{P}_{i})\approx 1$ for $i\ne j$. In cases where a candidate phrase has multiple matches, the confidence score is theoretically higher than any of the similarities $sim({p}_{c},{P}_{j})$. Thus, most of the data records are expected to have a confidence score that is equal to or higher than any of the similarity scores. Since records with similarity scores below ${\tau }_{sim}$ do not match to any centroid extraction patterns and are directly rejected, all similarity scores $sim({p}_{c},{P}_{j})$ are above ${\tau }_{sim}$. Therefore, we expect most records to have confidence scores above that of ${\tau }_{sim}$, which explains why there are few records falling into the category where the confidence score is less than ${\tau }_{sim}$. We conclude that setting ${\tau }_{c}$ above 0% provides no practical improvement in precision and recall; therefore, ${\tau }_{c}$ was fixed at 0% for this study.

In contrast, the minimum similarity threshold is a more practically useful performance parameter. While ${\tau }_{sim}$ is set to 90% during training, it can be tuned during data extraction to balance precision and recall. We evaluated the performance of ${\tau }_{sim}$ systematically by applying nine different ${\tau }_{sim}$ values to the articles of the evaluation set which were separated by 5% intervals; the results are shown in Fig. 2. As ${\tau }_{sim}$ is lowered from 95% to 55%, the precision decreases slightly while recall increases. The F-score has a peak value at ${\tau }_{sim}=65 \%$, where the Snowball parser has high precision (89%) and acceptable recall (51%). The F-score at this point is higher than that of AutoSentenceParser, and the Snowball model is thus considered to be a better choice for band gap information extraction. The reason why the minimum similarity threshold can noticeably affect performance is that ${\tau }_{sim}$ not only controls the shape (number and size) of sub-clusters, but it also implicitly controls the number of effective matches between a candidate phrase and the centroid extraction patterns. Lowering ${\tau }_{sim}$ means that new phrases have more matches to the centroid extraction patterns and they are more likely to be accepted, hence the increasing recall; but at the same time, more deviations from the centroid extraction patterns are allowed, hence the decreasing precision.

One interesting thing that we noticed is that about 4% of all testing records were correctly extracted by the Snowball model at ${\tau }_{sim}=85 \%$, but were missed out at ${\tau }_{sim}=65 \%$. This is, at first glance, counter-intuitive, since the recall at ${\tau }_{sim}=65 \%$ is nearly three times the recall at ${\tau }_{sim}=85 \%$, and one would expect a model with a lower threshold to cover all of the records found by the exact same model with a higher threshold. This is partially due to the bootstrapping feature of the Snowball model, that it updates itself when presented with new information. Whenever a new phrase is accepted, it is added to one of the sub-clusters, and the corresponding centroid pattern is updated, even at learning rate of 0. The new centroid pattern is now no longer guaranteed to successfully extract data from other candidate phrases that would be otherwise successfully extracted by the non-updated centroid extraction pattern. However, a more important origin of this behaviour lies in the data-extraction process. For a very high ${\tau }_{sim}$ value, a new phrase only matches to very few, if not just one, of the centroid extraction patterns. The match (or very rarely, matches) is then used to extract data from the candidate phrase, which is highly likely to be accurate. When ${\tau }_{sim}$ is lowered, a new phrase can match to multiple centroid extraction patterns, most of which are not perfectly suitable for the testing phrase. Having multiple potential centroid extraction patterns creates the possibility for the Snowball model to fit the candidate phrase to a non-best match, which will only produce less accurate, if not incorrect, results. Therefore, some of the true positive records become false positives when ${\tau }_{sim}$ decreases. Using less perfect matches for data extraction is also the primary reason why precision diminishes with decreasing ${\tau }_{sim}$.

Although the “disappearing” records do not affect the workflow or performance of the Snowball parser in unexpected ways, their presence indicates that there is performance headroom that cannot be achieved by simply tuning ${\tau }_{sim}$ to a single value. To solve this problem, we developed a new approach that we call “dynamic ${\tau }_{sim}$”. Data extraction is divided into two passes. In the first pass, ${\tau }_{sim}$ is set to a relatively high value (85% in this work), close to the value used in training. Thereby, if data records are found in a sentence, the records are accepted, and the second pass is skipped. If no data records are found, the second pass is executed, whereby the sentence is processed by another Snowball model with the exact same sub-clusters but with a lower ${\tau }_{sim}$ value (65% in this work). This new workflow can capture the “disappearing” records that would not be extracted by simply optimizing ${\tau }_{sim}$ to 65%, while avoiding the double counting problem caused by running two models independently, hence giving a performance boost over single-pass workflow. Evaluation shows that the double-pass workflow gives a 1% increase in precision and a 3% increase in recall. These results are also given at the end of Table 4.

Overall, a total of 100,236 records were compiled into the final database, 92,437 of which are extracted by the two parsers of ChemDataExtractor, and 7,656 are sourced from BandgapDB. The weighted precision of all records that are extracted by ChemDataExtractor is estimated to be 84%, and the weighted recall is 65%, giving an F-score of 73%. Thereby, a record randomly chosen from the NLP part of the database has an 84% probability of being correct, and the database covers 65% of all band gap records in the sourced papers. It is clear that by combining AutoSentenceParser and the Snowball model into a single pipeline, better performance can be achieved which is not possible for either one of the two parsers.

**Fig. 3:**
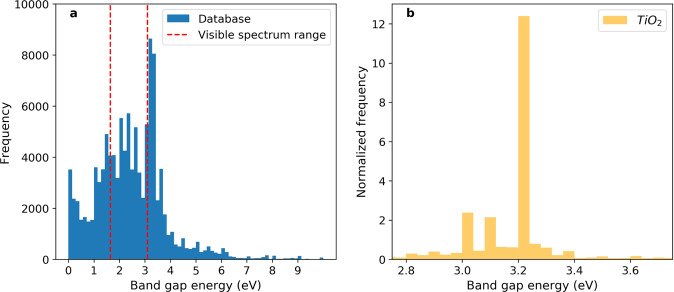
Band gap energy distribution of all records in the database (**a**) and titanium dioxide (**b**). Dashed red lines indicate the lower and upper bounds of visible spectrum at 1.65 eV and 3.10 eV.

Another way of validating the quality of the database is a visual inspection of the data. Figure 3 shows the band gap distribution of all data records in the database, as well as the band gap distribution for a typical transition metal oxide, TiO_2_, as a case study. The overall energy distribution of band gaps is centered around the visible spectrum range and 0 eV, where the peak at 3.4 eV results from two frequently mentioned compounds TiO_2_ and ZnO. This trend is in line with the general research interests in materials science: compounds that have unique transition properties (i.e. graphene can transit from a conductor to a semiconductor with band gap around 0 eV), and chemicals that can interact with solar light. For TiO_2_, there are three distinct peaks between 3.0 and 3.3 eV, which correspond to the three polymorphs of TiO_2_: anatase, rutile, and brookite. This again demonstrates the usefulness of the database, that users can acquire valuable information from the database with little background research.

## Usage Notes

The band gap database can be found in *Figshare*^27^. The database is configured in multiple formats for easier access to the data. The CSV and JSON formats are machine-readable and can be loaded using all mainstream programming languages including Python, C, Java, MatLab, and R. The MongoDB files require MongoDB to be pre-installed before use. Instructions on setting up MongoDB are available at https://docs.mongodb.com/manual/. The code used to generate the database can be easily repurposed to process more articles, by following the data-extraction pipeline outlined in https://github.com/QingyangDong-qd220/BandgapDatabase1.

The data entries can be queried by their attribute names, listed in Table 3. For example, chemical compounds can be queried by the raw text string that is extracted from the source document as well as the normalized compound name which is recognized by the Chemical Named Entity Recognition (CNER) function of ChemDataExtractor. The compounds can therefore be categorized into sub-classes so that further combination and comparison can be made. The user can also prioritize precision over recall to obtain a more accurate (cleaner) database, by removing data entries with lower confidence scores that are assigned by the modified Snowball pipeline. This improves precision at the cost of affording lower recall, as shown in Fig. 2.

## Data Availability

All source code for this work is freely available under the MIT license. The source code used to generate the band gap database is available at *Figshare*^27^ and https://github.com/QingyangDong-qd220/BandgapDatabase1. The updated patch for the modified Snowball algorithm, which is now compatible with nested models, is available at https://github.com/QingyangDong-qd220/BandgapDatabase1/tree/main/chemdataextractor2. A clean build of the ChemDataExtractor version 2.0 code is available at http://www.chemdataextractor2.org/download.
